# Acoustic Molecular Imaging Beyond the Diffraction Limit In Vivo

**DOI:** 10.1109/ojuffc.2022.3212342

**Published:** 2022-10-05

**Authors:** THOMAS M. KIERSKI, RACHEL W. WALMER, JAMES K. TSURUTA, JIANHUA YIN, EMMANUEL CHÉRIN, F. STUART FOSTER, CHRISTINE E. M. DEMORE, ISABEL G. NEWSOME, GIANMARCO F. PINTON, PAUL A. DAYTON

**Affiliations:** 1 Joint Department of Biomedical Engineering, UNC-Chapel Hill and NC State University, Chapel Hill, NC 27599 USA; 2 Sunnybrook Research Institute, Toronto, ON M4N 3M5, Canada; 3 Department of Medical Biophysics, University of Toronto, Toronto, ON M4N 3M5, Canada

**Keywords:** Molecular imaging, superharmonic imaging, ultrasound, ultrasound contrast agents, ultrasound localization microscopy

## Abstract

Ultrasound molecular imaging (USMI) is a technique used to noninvasively estimate the distribution of molecular markers *in vivo* by imaging microbubble contrast agents (MCAs) that have been modified to target receptors of interest on the vascular endothelium. USMI is especially relevant for preclinical and clinical cancer research and has been used to predict tumor malignancy and response to treatment. In the last decade, methods that improve the resolution of contrast-enhanced ultrasound by an order of magnitude and allow researchers to noninvasively image individual capillaries have emerged. However, these approaches do not translate directly to molecular imaging. In this work, we demonstrate super-resolution visualization of biomarker expression *in vivo* using superharmonic ultrasound imaging (SpHI) with dual-frequency transducers, targeted contrast agents, and localization microscopy processing. We validate and optimize the proposed method *in vitro* using concurrent optical and ultrasound microscopy and a microvessel phantom. With the same technique, we perform a proof-of-concept experiment *in vivo* in a rat fibrosarcoma model and create maps of biomarker expression co-registered with images of microvasculature. From these images, we measure a resolution of 23 μm, a nearly fivefold improvement in resolution compared to previous diffraction-limited molecular imaging studies.

## I. INTRODUCTION

GIVEN the diversity of neoplastic diseases and their potential to develop into life-threatening conditions, there is a need for safe and effective characterization of tissues across many applications. Assessment of disease biomarkers, such as the vascular “fingerprint” of tumors and their associated microenvironment, is infeasible with many biomedical imaging techniques and typically requires terminal pathology studies. One hallmark of cancer is the deregulation of angiogenic signaling, which results in a chaotic and densely-packed network of blood vessels around the growth [1], [2]. Ultrasound imaging is a good candidate for assessing cancers *in vivo* because it provides excellent spatial and temporal resolution, does not expose patients to ionizing radiation, and is substantially less expensive and more accessible compared to modalities such as magnetic resonance imaging and positron emission tomography. In addition to imaging anatomical structures, ultrasound may also be used to measure the mechanical properties of tissues [3] as well as the velocity of blood flow [4] in real-time. The clinical utility and versatility of biomedical ultrasound is further increased by microbubble contrast agents (MCAs). MCAs are typically composed of a heavy gas core and a lipid, protein, or polymer shell, with diameters between approximately 1 and 10 μm. When administered intravenously, these contrast agents serve as blood pool markers. Contrast-enhanced ultrasound imaging (CEUS) has many applications in the context of cancer, such as quantifying perfusion in suspicious lesions [5], [6].

With ultrasound molecular imaging (USMI), it is also possible to visualize biomarker expression *in vivo* by imaging MCAs that have been modified to interact with a specific vascular target. Microbubbles can be functionalized for USMI by the addition of one or more ligands to the shell architecture [7]. When targeted bubbles are introduced into circulation, they flow until binding at the site of interest. In comparison, non-targeted microbubbles flow freely throughout the vasculature. Some notable applications of USMI include early detection of cancer [8], classifying breast lesions [9], quantifying inflammation [10], and monitoring response to treatments [11], [12], [13], [14].

Differential targeted enhancement (dTE) is a common approach for estimating the distribution of contrast agent binding *in vivo* [7], [15], [16], [17]. For this method, targeted MCAs are injected and allowed to circulate for a predetermined length of time to facilitate microbubble targeting and clearance of residual unbound contrast. An image is acquired at this point, after which microbubbles in the field of view are disrupted with a high-amplitude ultrasound transmission. Some time is given for any remaining bubbles to reperfuse the field of view, after which an additional image is collected. The difference between the pre- and post-disruption images provides an estimate of contrast agent binding over a region of interest. While this method is effective for estimating the amount of targeting within a tumor, its resolution is limited by diffraction. Many diagnostically relevant structures exist at spatial scales smaller than the point spread function (PSF) of a standard clinical imaging system (*e.g.*, capillaries from tumor-associated angiogenesis). Alternative approaches for assessing bound MCAs have been proposed, such as those based on dwell time [14], normalized singular spectrum area [18], [19], and convolutional neural networks [15].These techniques are promising real-time methods for estimating molecular expression. However, similar to dTE, the resolutions of these methods are largely governed by diffraction.

Ultrasound localization microscopy (ULM) is a super-resolution imaging technique analogous to optical localization methods [20], [21], [22], [23] and improves resolution by an order of magnitude [24], [25], [26] enabling noninvasive imaging of capillaries. Briefly, a bolus of MCAs is administered intravenously, and a large sequence of images is collected (normally N > 1000). From these images, individual microbubbles are isolated from the background speckle and localized onto a super-resolution grid. These localizations accumulate over the full set of images to produce a map of the underlying vessel structure that is much finer than the PSF of the imaging system. To date, a fundamental limitation of ULM has been that most methods for isolating MCAs from tissue before localization rely on the spatiotemporal decorrelation that results from MCAs flowing through the circulatory system while the sequence of images is acquired. However, if a microbubble is bound to an endothelial target, it is in a zero-velocity state relative to the nearby tissue. Hence, spatiotemporal filtering approaches are not feasible for imaging stationary MCAs, such as those in USMI.

In contrast to biological tissues, microbubble contrast agents are resonant structures which oscillate with broadband harmonics when excited by a sound wave near their resonant frequency. Thus, MCAs can be detected spectrally instead of spatiotemporally. Superharmonic imaging (SpHI) is a technique that takes advantage of this phenomenon by recording the higher harmonics (*i.e.*, superharmonics) of the transmitted frequency to create a high-resolution image nearly devoid of tissue speckle [27], [28], [29], [30].

Notably, superharmonic generation is not influenced by the velocity of MCAs [31], resulting in excellent contrast-to-tissue ratio even for stationary bubbles. Until recently, SpHI had not been applied to ULM because commercially available ultrasound transducers do not have the bandwidth necessary for receiving echoes beyond the third harmonic of the transmitted pulse. Our team recently developed a multifrequency linear array transducer for plane-wave SpHI [32] and demonstrated its high sensitivity to MCAs independent of their velocity [31]. In this work, we combine this dual-frequency transducer with targeted MCAs, ULM processing, and microbubble tracking, to achieve for the first time super-resolution imaging of molecularly bound contrast agents *in vivo*. We provide a description and validation of this novel method for super-resolution USMI using superharmonic ultrasound localization microscopy to produce co-registered maps of microvessels and angiogenic signaling.

## II. MATERIALS AND METHODS

### A. CONTRAST AGENT PREPARATION

Our in-house non-targeted microbubble contrast agent was formulated from a 1mM lipid solution that contained 900 *μ*M 1,2-distearoyl-sn-glycero-3-phosphocholine (DSPC) and 100 *μ*M 1,2-distearoyl-sn-glycero-3-phosphoethanolamine-N-[methoxy(polyethylene glycol)-2000] (DSPE-PEG2000) lipids in 5% (v/v) glycerol and 15% (v/v) propylene glycol in phosphate-buffered saline (PBS). A biotinylated microbubble contrast agent (4.5 mole %) was formulated by replacing 45 *μ*M of the DSPE-PEG2000 in the in-house formulation with 1,2-distearoyl-sn-glycero-3-phosphoethanolamine-N-[biotinyl(polyethylene glycol)-2000] (DSPE-PEG2000-biotin) (Avanti Polar Lipids, Alabaster, AL, USA). A cRGD microbubble contrast agent (0.4 mole %) targeted to bind to *α*_*v*_*β*_3_ integrin was formulated from our in-house lipid solution supplemented with 4 *μ*M synthetic azide-activated cycloArg-Gly-Asp (cRGD) peptide (Peptides Int’l, Louisville, Kentucky, USA) conjugated to DSPE-PEG2000-DBCO lipid via click chemistry. The size distributions and concentrations of each contrast agent were measured using an Accusizer FX-Nano (Entegris, Billerica, MA, USA). Table 1 contains a summary of each MCA formulation.

### B. DUAL-FREQUENCY SYSTEM DESCRIPTION

**FIGURE 1.**
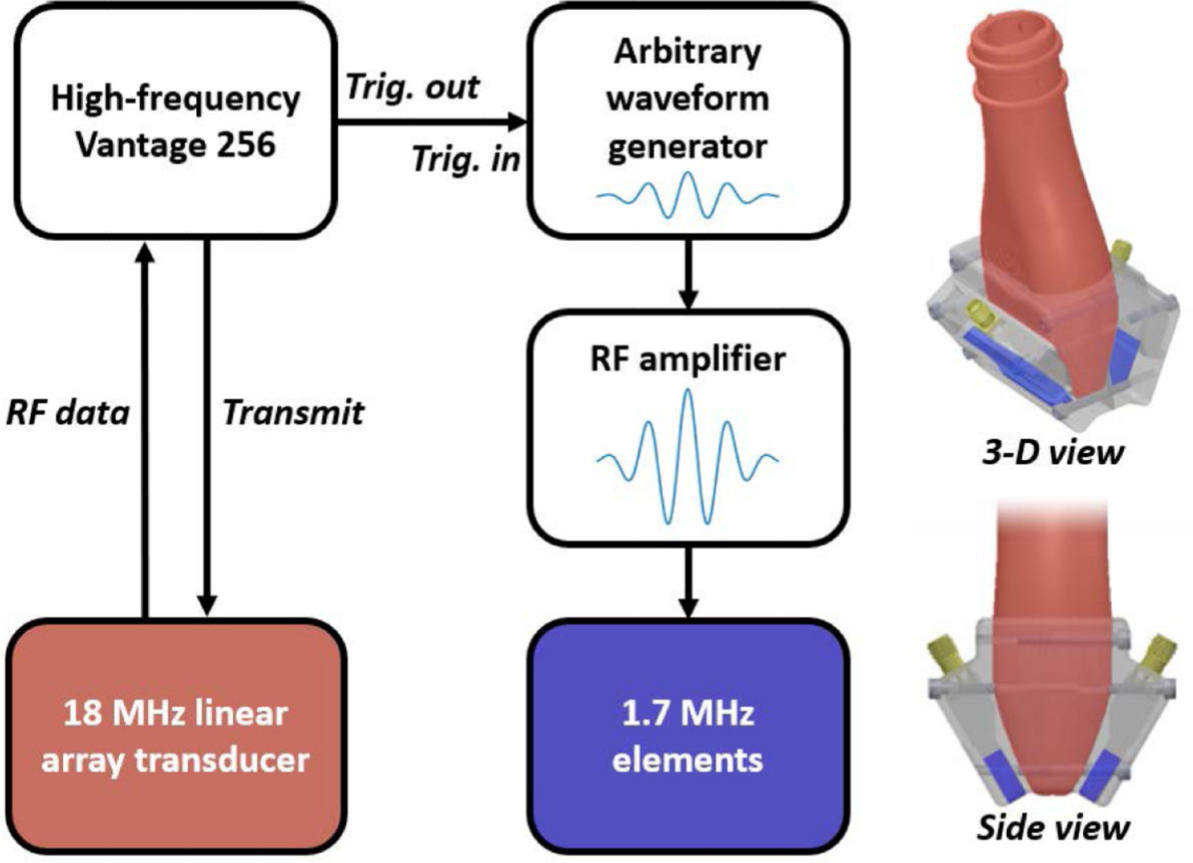
A diagram of the dual-frequency system with a color-coded rendering of the transducer. In this representation, arrows denote the direction of signal flow. The transducer is composed of an 18 MHz linear array (red) and two additional 1.7 MHz elements (blue). The low-frequency elements are held in place on either side of the linear array using a custom 3-D printed bracket. The linear array is operated normally for B-mode imaging, and superharmonic imaging is performed by transmitting with the low-frequency elements and receiving with the high-frequency array. All radiofrequency data is digitized and recorded with a high-frequency Vantage 256 scanner.

The dual-frequency transducer characterized previously by Cherin and colleagues was used for imaging experiments [31], [32]. Briefly, it consisted of an 18 MHz linear array (MS-250, VisualSonics, Toronto, Canada) outfitted with two additional 1.7 MHz transducers (Fig. 1). The low-frequency elements are arranged symmetrically about the axial-lateral plane of the high-frequency array, and their lateral size is equal to the 22.5 mm aperture of the high-frequency array. Transmissions from the two elements interfere and produce a main lobe for exciting contrast agents at depths ranging between approximately 16 and 27 mm (see [31] and [32] for images of simulated and experimental beam plots).

This device is capable of conventional B-mode imaging (transmitting and receiving using the linear array) and superharmonic imaging (transmitting using the low-frequency elements, receiving using the linear array). For superharmonic imaging, a one-cycle 1.7 MHz pulse from an arbitrary wave-form generator (AWG2021, Tektronix, Beaverton, OR, USA) was amplified by 50 dB (240 L radiofrequency amplifier, ENI, Rochester, NY, USA) to drive the two low-frequency elements. For B-mode imaging, the linear array transducer was driven with a two-cycle 15.625 MHz pulse from a high-frequency Vantage 256 (Verasonics, Kirkland, WA, USA). For both imaging modes, all transmissions were unfocused and unsteered. Radiofrequency data from the high-frequency array were recorded with a sampling frequency of 62.5 MHz and bandpass filtered (15.625 MHz center frequency, 66% bandwidth). SpHI and B-mode imaging were performed at mechanical indices (MI) of 0.24 and 0.11, respectively (measured in water using HNA-0400, Onda Corporation, Sunnyvale, CA, USA). Volumetric imaging *in vivo* was performed using a motion stage (XSlide, Velmex, Inc., Bloomfield, NY, USA) controlled by a custom LabVIEW program (National Instruments, Austin, TX, USA) to translate the probe in the elevational dimension.

### C. BASELINE RESOLUTION MEASUREMENT FOR THE HYBRID DUAL-FREQUENCY ARRAY

Similar to our previous work [33], we created a very dilute suspension of microbubble contrast agents by adding 1 .3e6 microbubbles to a 4-liter tank of distilled water. A stir plate (Thermolyne Cimarec, Barnstead International, Dubuque, IO, USA) set to its lowest speed was used to keep the solution well-mixed. This suspension was imaged using the dual-frequency system at 10 frames per second (fps) and MI = 0.24, with the frame rate chosen to guarantee independent realizations of bubbles across different images. Data were collected for 20 seconds, resulting in 200 unique images. Delay-and-sum beamforming was performed offline with F# = 2 at depths between 17 mm and 27 mm, and lateral positions between ± 7.5 mm. Axial and lateral profiles were automatically extracted from microbubbles in the beamformed images using a custom MATLAB routine, and the full-width at half-maximum (FWHM) values were measured.

### D. DATA COLLECTION FOR IN VITRO BINDING EXPERIMENT

A cellulose tube (200 μm diameter) was coated with a solution of 5 mg/mL avidin in PBS at room temperature and stored at 4°C for 16 hours. The tube was then submerged in a tank of water in the field of view of an inverted microscope (IX71, Olympus, Tokyo, Japan) using a 60× immersion lens. The microscope was connected to a high-speed camera (FASTCAM SA1.1. Photron, Tokyo, Japan) that digitized images on a 1024×1024 grid at 250 fps. The microscope was focused on the upper wall of the cellulose tube, and B-mode imaging was utilized to position the ultrasound scanner.

For all imaging experiments related to tuning the molecular localization algorithm, a contrast agent was diluted to 1e8 mL^−1^ in PBS and injected into the tube. The bubbles were allowed to float for 3 minutes for binding to occur, after which a volume flow rate of 10 μLmin^−1^ was imposed with a syringe pump (Harvard Apparatus, Holliston, MA, USA). A video was captured at the beginning of the flow to assess contrast-avidin binding. After one minute of flow, 500 superharmonic ultrasound images were collected at 250 fps. Control and targeted imaging were performed using in-house bubbles and biotinylated bubbles, respectively.

### E. ANIMAL CARE AND IN VIVO DATA COLLECTION

*In vivo* imaging was performed on female Fischer 344 rats (Charles River Laboratories, Durham, NC, USA), and all imaging protocols were approved by the Institutional Animal Care and Use Committee at the University of North Carolina at Chapel Hill. Animals were housed in a cage measuring 140 in^2^ (individually-ventilated with a static micro-isolator) located within a vivarium with a simulated day-night cycle. Animals received regular daily monitoring and could freely access water and standard rat feed. At the time of imaging, all animals were between 11–12 weeks old and each weighed between 145 and 155 grams.

The fibrosarcoma (FSA) model was prepared as described previously [34], [35]. Small volumes of FSA tissue between 1–2 mm^3^ from a donor animal were transplanted into the right flanks of three rats, and tumors were allowed to grow for 10 days before imaging. For imaging experiments, animals were anesthetized with vaporized isoflurane mixed with oxygen, and the target area was shaved. A 24-gauge catheter was placed in the tail vein for administering the microbubble contrast agents.

For all animals, a volumetric B-mode scan was acquired before contrast imaging for anatomical reference. Then, a 100 μL bolus of 1e8 cRGD-labeled MCAs was injected via tail-vein catheter, followed by a two-minute wait to allow the bubbles to circulate and bind. Afterward, sets of 500 SpHI frames (250 fps) were acquired at 11 elevational positions spaced by 1 mm. After the targeted scans, data for a background ULM image were acquired at each elevational position. For each slice, a 100 μL bolus of 1e8 non-targeted MCAs was injected, and 25,000 superharmonic images were immediately acquired at 250 fps with B-modes interleaved.

As mentioned previously, SpHI suppresses tissue speckle, making it difficult to estimate physiological motion from the contrast-enhanced images. To overcome this challenge, B-mode acquisitions were interleaved between SpHI frames with a 1:1 ratio for all *in vivo* experiments, a technique which has previously been described [31], [36], [37]. This imaging sequence was implemented by alternating between low- and high-frequency transmissions. Tissue displacements were estimated by performing speckle tracking on the B-mode images as described in a later section. Note that the frame rate reported for each experiment corresponds to the time between consecutive SpHI frames (*i.e.*, the true pulse repetition frequency is doubled when accounting for the additional B-mode acquisitions).

### F. LOCALIZATION MICROSCOPY PROCESSING

For ULM processing, all SpHI images were beamformed offline using delay-and-sumona10-μm grid. Each image was thresholded at three times the noise floor (empirically determined), after which bubbles were localized by convolving with a Gaussian aperture calibrated to the PSF of the imaging system and detecting local maxima. For *in vivo* datasets, inter-leaved B-mode images were beamformed on a 10-μm grid, and a 1 mm × 1 mm region of interest (ROI) beneath the skin was manually selected for each elevational position. Speckle tracking with a normalized cross-correlation (NCC) search was performed on these ROIs to estimated is placement during image acquisition. B-modes with estimated displacements less than 100 μm and NCC values greater than or equal to 0.95 were used to correct localization coordinates from their corresponding SpHI frames, and the remaining localizations were discarded.

After motion correction (*in vivo* data only), bubbles were tracked between frames using the Hungarian algorithm (*simpletracker*, MATLAB). For the molecular targeting data, the max linking distance between frames was set to one pixel, and only tracks with a length of at least 12 frames (48 milliseconds) were considered. The minimum of 12 frames was determined to be sufficient for filtering out moving bubbles while ensuring that bound bubbles were not deflated from repeated sonication.

**FIGURE 2.**
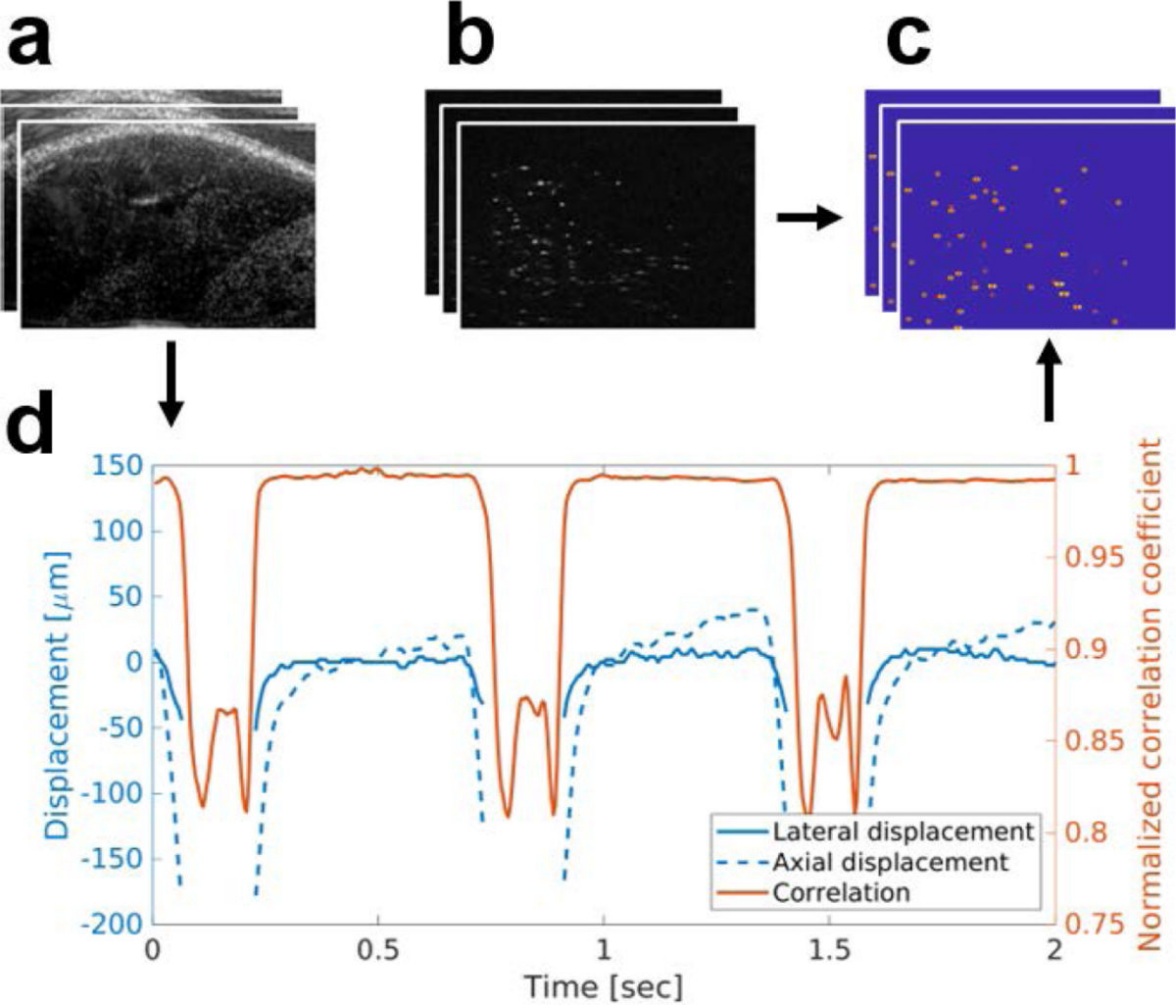
A flowchart outlining the steps for molecular ultrasound localization microscopy. (a) B-mode images are interleaved between (b) superharmonic contrast images. (c) Microbubbles are detected in the contrast images and localized. (d) Displacements are measured from the B-modes, and used to correct the coordinates in (c). Motion correction is only performed for *in vivo* imaging. A microbubble is considered bound if it persists locally (after motion correction, if applicable) for 12 or more consecutive time points.

Tracks with a final coordinate located within one pixel of the first tracked coordinate were considered to be bound bubbles. This threshold of a single pixel was deemed appropriate to prevent filtering out bubbles whose positions might seemingly fluctuate as a result of localization error. The first index of each of these tracks was used as the coordinate of the bound MCA. A summary of the molecular imaging processing is provided in Fig. 2. For background ULM images, the max linking distance between frames was set to 100 μm, which corresponds to a maximum velocity of 25mms^−1^. Only tracks with lengths greater than or equal to 10 frames were considered to reduce noise in the rendered images. The tracks were accumulated to generate the final images. Molecular and background ULM images were co-registered using their respective reference B-mode frames used for motion tracking. Volumetric images were rendered in 2-D using a maximum intensity projection.

### G. OPTICAL - ULTRASOUND CALIBRATION

To ensure that the output of the ULM system scales linearly with the true local density of microbubbles, we also performed a calibration by comparing MCA counts in optical and ultrasound images across various concentrations of non-targeted microbubbles. Using the experimental setup described in section II-B, we collected concurrent optical and ultrasound videos of dilutions of non-targeted contrast agents with concentrations between 5e5 and 1e7 mL^−1^ flowing through the tube at 5 μLmin^−1^. The output of the inverted microscope was digitized with a DSLR camera (X-T2, Fuji-film, Tokyo, Japan) on a 1920 × 1080 pixel grid at a rate of 30 fps. Ultrasound radiofrequency data were collected at 100 fps and beamformed with a pixel size of 10 μm.

Microbubbles were counted in the ultrasound images using the techniques described in section II-F. The optical images were processed as follows:

1) An 8th order bandpass Butterworth filter with low and high frequency cutoffs of 0.75 and 29 Hz, respectively, was applied to each pixel of the optical data to remove the static background and some high-frequency noise.
2) Each pixel was replaced with its absolute value and then thresholded to 3× the noise floor (empirically measured).
3) The cube root of each pixel was taken to compress the dynamic range of the images.
4) Circles in each frame were detected using a multiscale Hough transform (MATLAB function *imfindcircles*).

Bubble counts were divided by the areas of the tube visible for each imaging modality to convert the raw counts to densities. An ordinary least squares linear model was fit to the data using the *statsmodels* Python package v0.13.

### H. VESSEL CENTERLINE EXTRACTION AND ANALYSIS

Vessel centerlines were retrieved from each 2-D ULM image using Aylward and Bullitt’s algorithm [38]. Vessels were processed in 2-D because of the large elevational step size between each slice. The distance metric (DM) is equal to the total path length of each centerline divided by the straightline distance between the vessel endpoints. For a centerline composed of *n* points with coordinates $p_{x}$,

(1) $$\text{DM}=\frac{\sum_{x=1}^{n-1}\left\|p_{x}-p_{x+1}\right\|}{\left\|p_{1}-p_{n}\right\|}.$$

The sum-of-angles metric (SOAM) is equal to the summation of angles between consecutive triplets of points, divided by the total path length of the centerline. For each triplet of points composing two vectors, $v_{x}$ and $v_{x+1}$,

(2) $$\text{SOAM}=\frac{\sum_{x=1}^{n-2}\cos^{-1}\left(\frac{v_{x}}{\left\|v_{x}\right\|}\cdot \frac{v_{x+1}}{\left\|v_{x+1}\right\|}\right)}{\sum_{x=1}^{n-1}\left\|p_{x}-p_{x+1}\right\|}.$$

For each vessel in each image, the distance to the nearest molecular localization was computed using the *dsearchn* function (MATLAB, MathWorks, Inc.). Outliers were determined using the *isoutlier* function in MATLAB on the full set of DM and SOAM values, and a vessel was removed if it was an outlier for either tortuosity metric.

### I. MEASURING THE RESOLUTION OF ULM IMAGES WITH FOURIER RING CORRELATION

Fourier ring correlation (FRC) is a robust, automatic method for measuring the resolution of images, such as those captured with super-resolution microscopy [40]. FRC measures the resolution of an imaging system by quantifying the agreement in the frequency domain between two images of the same scene with independent noise realizations. This is accomplished by computing the normalized cross correlation between sets of concentric rings (or shells for 3-D images) in Fourier space to determine the spatial frequency beyond which true structures and noise are indiscernible. The correlation between the *i*th frequency bins from the two images is given by

(3) $$\mathrm{FRC}\left(r_{i}\right)=\frac{\sum_{r\in r_{i}}F_{1}(r)\cdot F_{2}{\left(r\right)}^{*}}{\sqrt{\sum_{r\in r_{i}}F_{1}^{2}(r)\cdot \sum_{r\in r_{i}}F_{2}^{2}(r)}},$$

where $r_{i}$ is the *i*th frequency bin, and *F*_1_ and *F*_2_ are the Fourier transforms of the two images. To measure the resolution of our ULM imaging system, we performed the one-image FRC approach described by Koho and colleagues [39], utilizing the open-source repository linked to said publication. The one-image method splits a single image into four independent images, and the average of two FRC measurements is reported. The numeric resolution corresponds to the spatial frequency at which the correlation curve drops below $\frac{1}{7}$ [39]. Our measurement is performed on the background localization microscopy image slice from the center of the tumor (*i.e.*, containing blood vessels) shown in Fig. 7h, since the molecular localization maps are sparse, binary images. We assume that since we are using the same imaging platform, beamforming, and localization algorithms for the background and molecular data, the resolutions of the two approaches should be equal.

### J. ESTIMATING THE DEGREE OF VESSEL RECONSTRUCTION

The degree of vessel reconstruction in ultrasound localization microscopy images was estimated using the methods presented by Dencks et al., who model the accumulation of localizations as a zero-inflated Poisson process [41]. For each of the three tumors imaged during the study, we drew regions of interest (ROIs) around the tumor boundary for each slice of the volume. The degree of reconstruction (DOR) was estimated for each ROI using the equation

(4) $$\text{DOR}=1-e^{-\hat{\Lambda }},$$

where $\hat{\Lambda }$ is given by

(5) $$\hat{\Lambda }=W_{0}\left(\frac{-T_{2}}{T_{1}}e^{-\frac{T_{2}}{T_{1}}}\right)+\frac{T_{2}}{T_{1}}.$$

*T*_1_ is equal to the number of non-zero pixels within the ROI, *T*_2_ is equal to the sum of the counts of the non-zero pixels within the ROI, and *W*_0_(*x*) is the main branch of Lambert’s W-function (MATLAB function *lambertw*).

## III. RESULTS

### A. IN VITRO STUDIES

#### 1) VALIDATION OF MOLECULAR ULM ALGORITHM PARAMETERS

**FIGURE 3.**
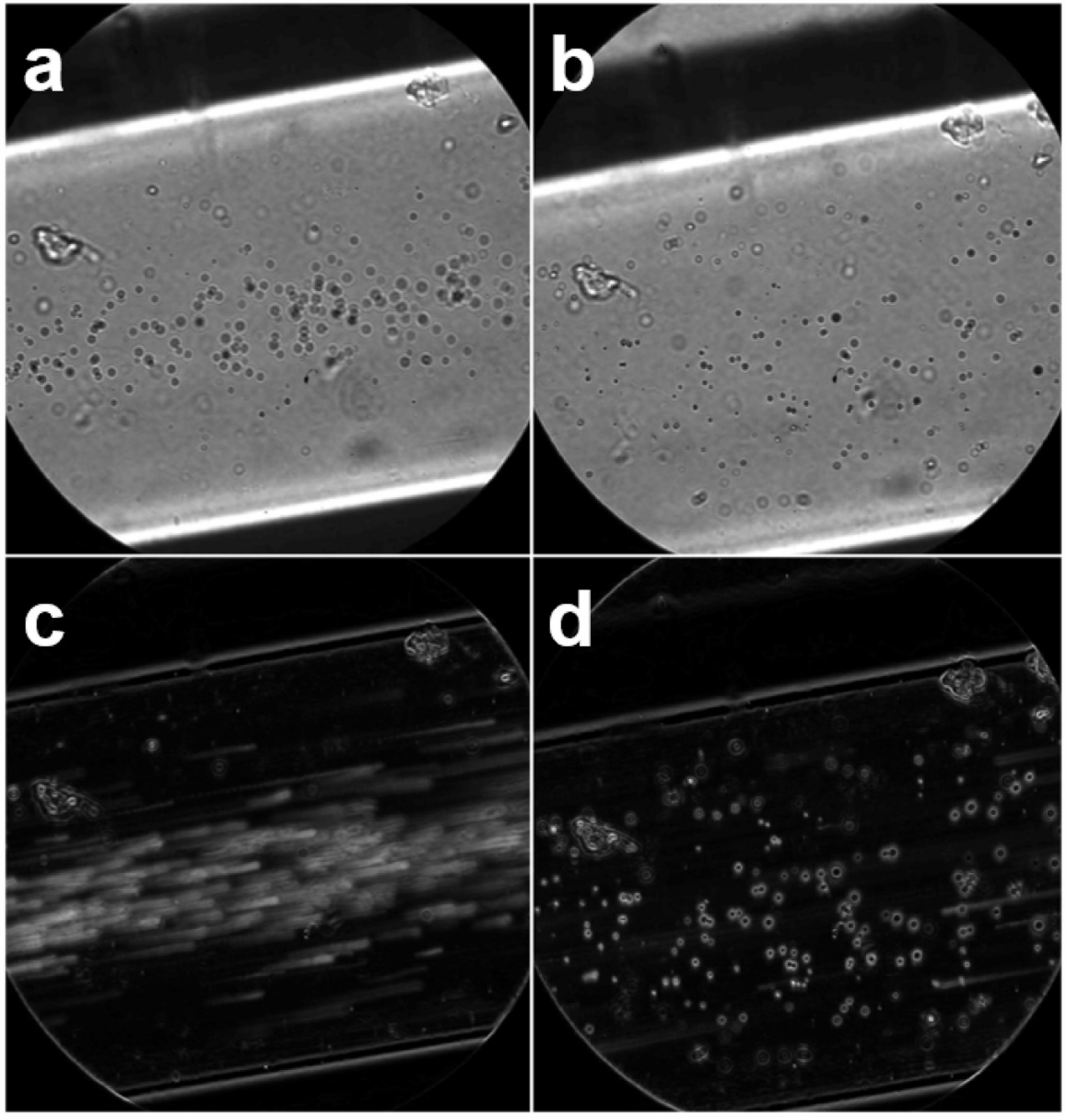
Optical comparison of control and biotinylated contrast agents in a microvessel phantom coated with avidin. *Top row*: bubbles near the upper wall of the tube after 3 min of flotation for control (a) and biotin (b) trials. *Bottom row*: Standard deviation images generated from 1 second of optical data captured after introducing flow of saline for control (c) and biotinylated (d) microbubbles. Streaks in the standard deviation image result from bubble movement.

**FIGURE 4.**
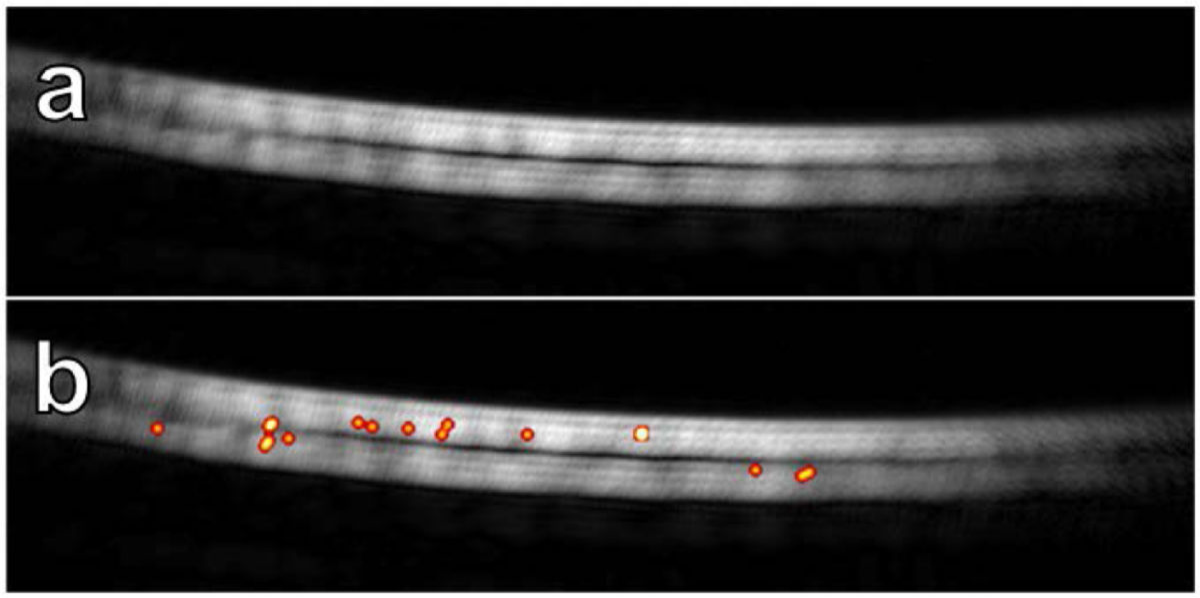
Example super-resolution molecular images from control and targeted trials in a microflow phantom. (a) Processing data for the unmodified (control) contrast agent yields no detections. (b) The interaction between the biotinylated microbubbles and the avidin coating produces numerous localizations after processing (localizations are blurred for visualization only).

We first demonstrated the feasibility of super-resolution USMI using concurrent optical and ultrasonic imaging of control and targeted (biotinylated) MCAs in an avidin-coated microflow phantom. Optical microscopy revealed that targeted microbubbles adhered to the vessel wall and were retained during flow after a few initial loose bubbles dislodged, whereas control bubbles exhibited no retention (Fig. 3). We also demonstrated that the molecular localization algorithm described earlier (Section II-F) was not sensitive to free-flowing control contrast agents but detected the biotinylated bubbles, which were bound to the walls of the tube (Fig. 4).

#### 2) OPTICAL - ULTRASOUND CALIBRATION

**FIGURE 5.**
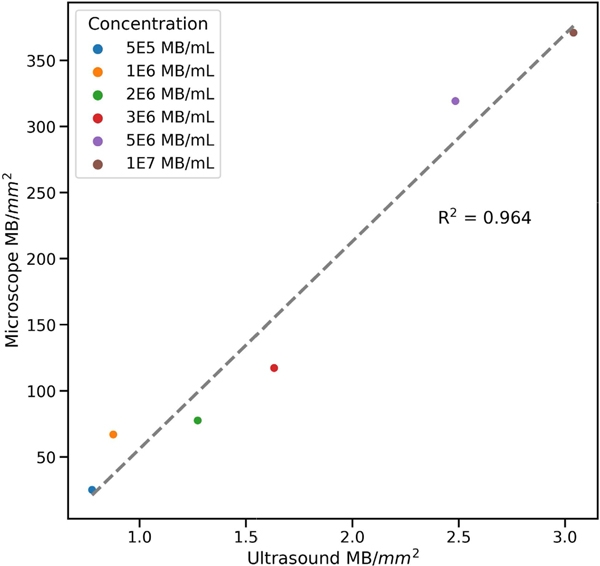
Measuring the correlation between optical and dual-frequency ultrasound bubble counts. Counting bubbles in optical and ultrasound videos across a range of different MCA concentrations suggests that the ultrasound bubble count scales linearly with respect to the ground truth bubble density.

By counting microbubbles in the ultrasound and optical videos, we measured the relationship between acoustic and optical bubble densities. Fitting an ordinary least squares linear model to the data yields *R*^2^ = 0.964, suggesting a strongly linear relationship between the output of the ultrasound imaging and the true local density of microbubbles (Fig. 5). We tested higher concentrations of microbubbles, but we limited our analysis to the range of concentrations for which the optical counting method was robust (*i.e.*, not too many overlapping bubbles for the Hough transform). It is important to note that because the dual-frequency ultrasound system is only sensitive to a small fraction of the polydisperse bubble population, it is possible to image higher concentrations with ultrasound (up to approximately 1e8 mL^−1^) while maintaining the necessary sparsity for accurate localizations.

#### 3) RESOLUTION MEASUREMENTS

**FIGURE 6.**
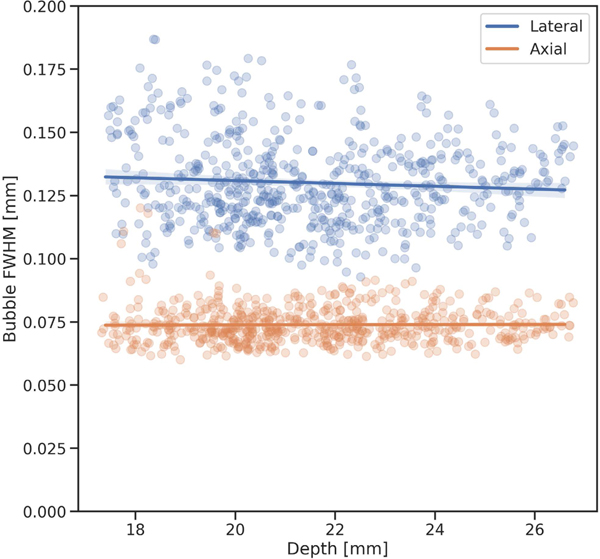
Resolution measurements of the dual-frequency transducer in SpHI mode. The original resolution of the superharmonic imaging device was empirically determined by measuring the point spread function repeatedly within a region of interest over 400 independent images of bubbles floating in a water tank. The mean axial and lateral FWHM values were 73 ± 7 μm and 130 ± 17 μm, respectively.

The superharmonic point spread function was repeatedly measured *in vitro* with the same mechanical index of 0.24 used elsewhere in the study. The mean axial and lateral FWHM values were 73 ± 7 μm and 130 ± 17 μm, respectively (Fig.6).As expected, given the constant F#used during beamforming, there was no change in the average FWHM vs. axial depth.

### B. IN VIVO IMAGING

#### 1) TUMOR IMAGES AND RESOLUTION MEASUREMENT

**FIGURE 7.**
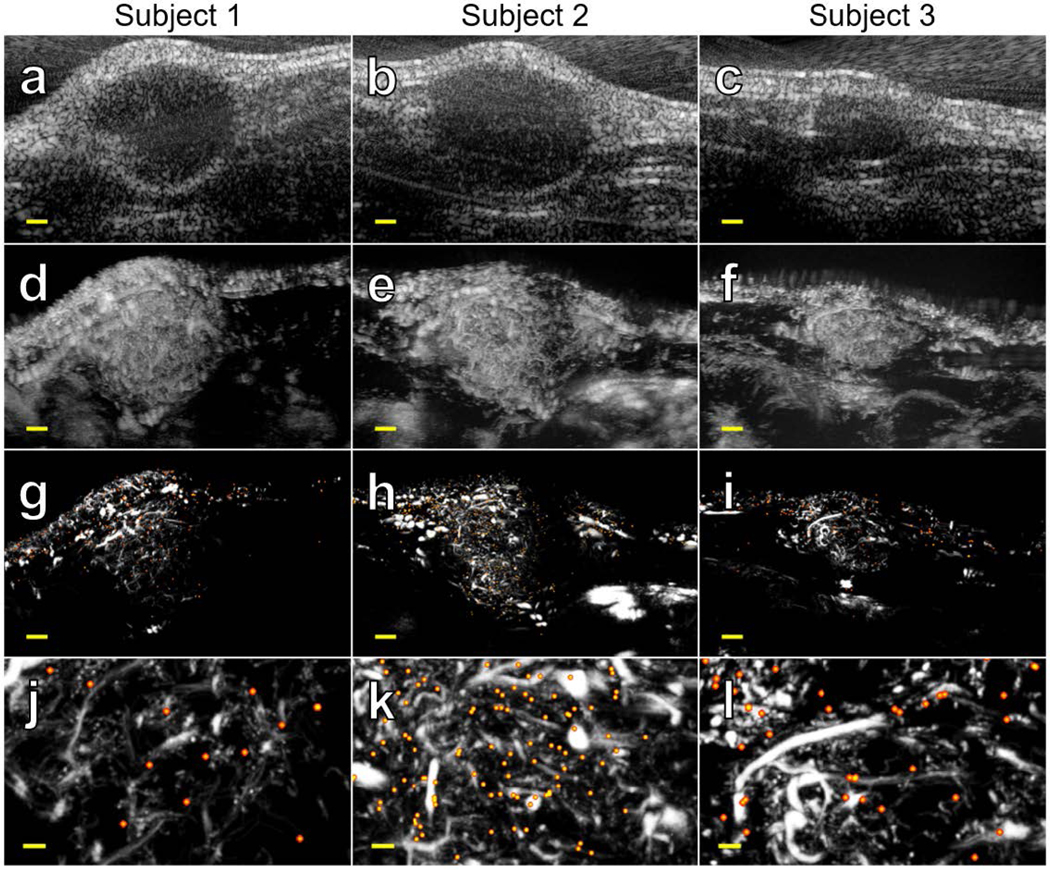
Super-resolution ultrasound molecular imaging in three rodent fibrosarcomas. (a-c) B-mode (standard ultrasound) images from the center of each tumor captured with the high-frequency elements of the dual-frequency array (dynamic range = 40 dB). (d-f) Maximum intensity projections generated from dual-frequency superharmonic images acquired across the tumor volumes (dynamic range = 30 dB). (g-i) Maximum intensity projections created with superharmonic ultrasound localization microscopy (gray colormap) with super-resolved molecular signaling overlaid (warm colormap, localizations are blurred to improve visibility, true size is smaller). Scale bars are 1 mm. (j-l) 2 mm x 3.5 mm selections from each ULM image showing microvascular and biomarker detail. Scale bars are 250 μm. Images in the same column are the same tumor.

**FIGURE 8.**
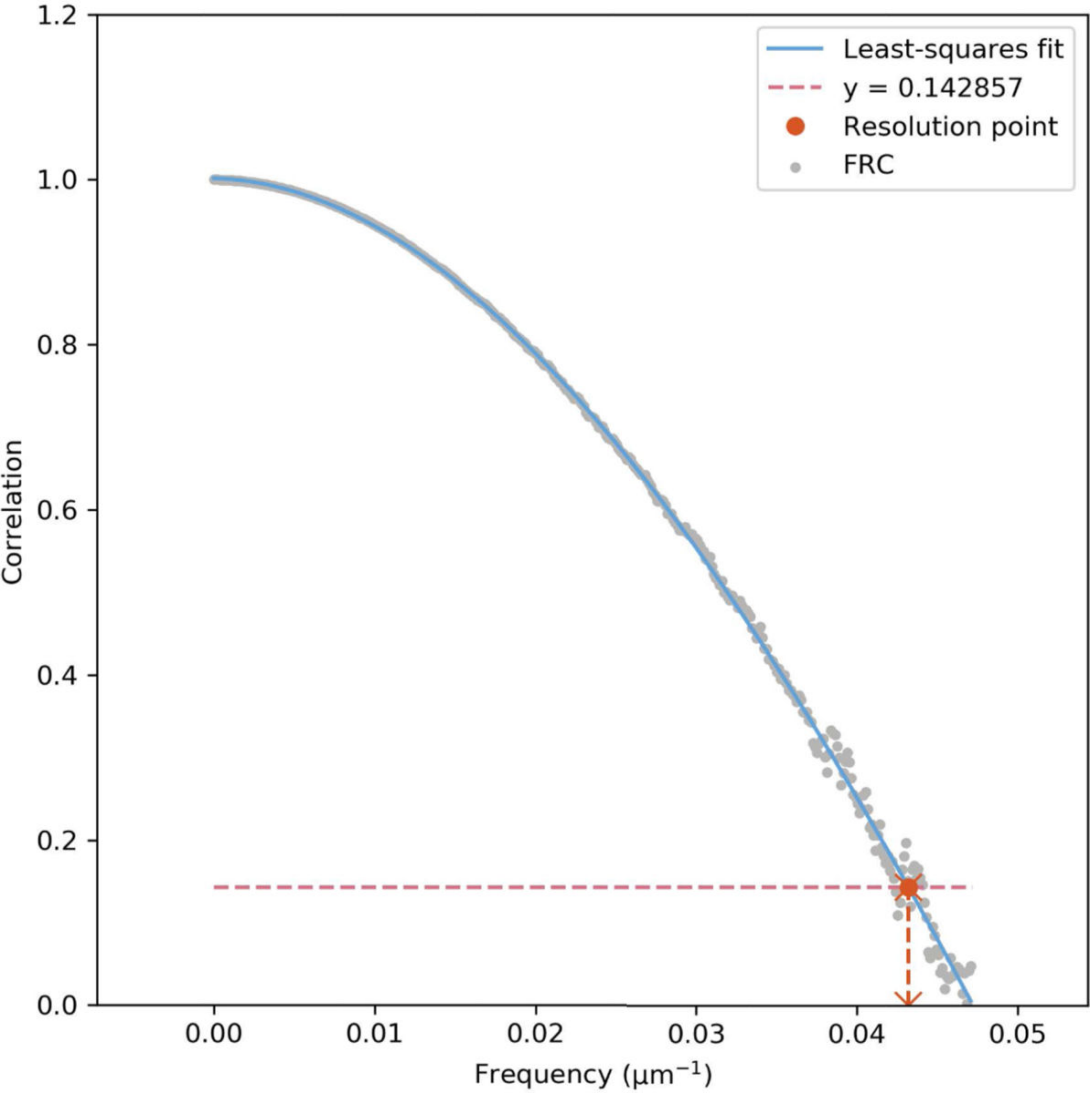
Fourier ring correlation plot for ULM image of rat fibrosarcoma vasculature. Single-image FRC plot corresponding to the centermost microvascular image from the center of the tumor in Fig. 7b. This plot was created using the open-source library linked to the publication by Koho and colleagues [39].

B-mode images of the rodent fibrosarcoma tumors revealed diameters ranging between 4 and 9mm (Fig.7a-c). Maximum intensity projections of the superharmonic data confirmed that each tumor was well-vascularized, but the diffraction-limited resolution of the scanner limited the separability of individual vessels (Fig. 7d-f). By overlaying the output of the molecular localization algorithm on the super-resolution image of the same tumor, it was possible to visualize microvessels and biomarker expression at a scale beyond the diffraction limit (Fig. 7g-l), with a resolution of 23 μm as measured by Fourier ring correlation (Fig. 8). This result is a nearly fivefold improvement in resolution compared to the previously described molecular acoustic angiography [42], and a threefold improvement compared to the axial resolution of the ultrasound system in SpHI mode (Fig. 6).

#### 2) VESSEL METRICS

**FIGURE 9.**
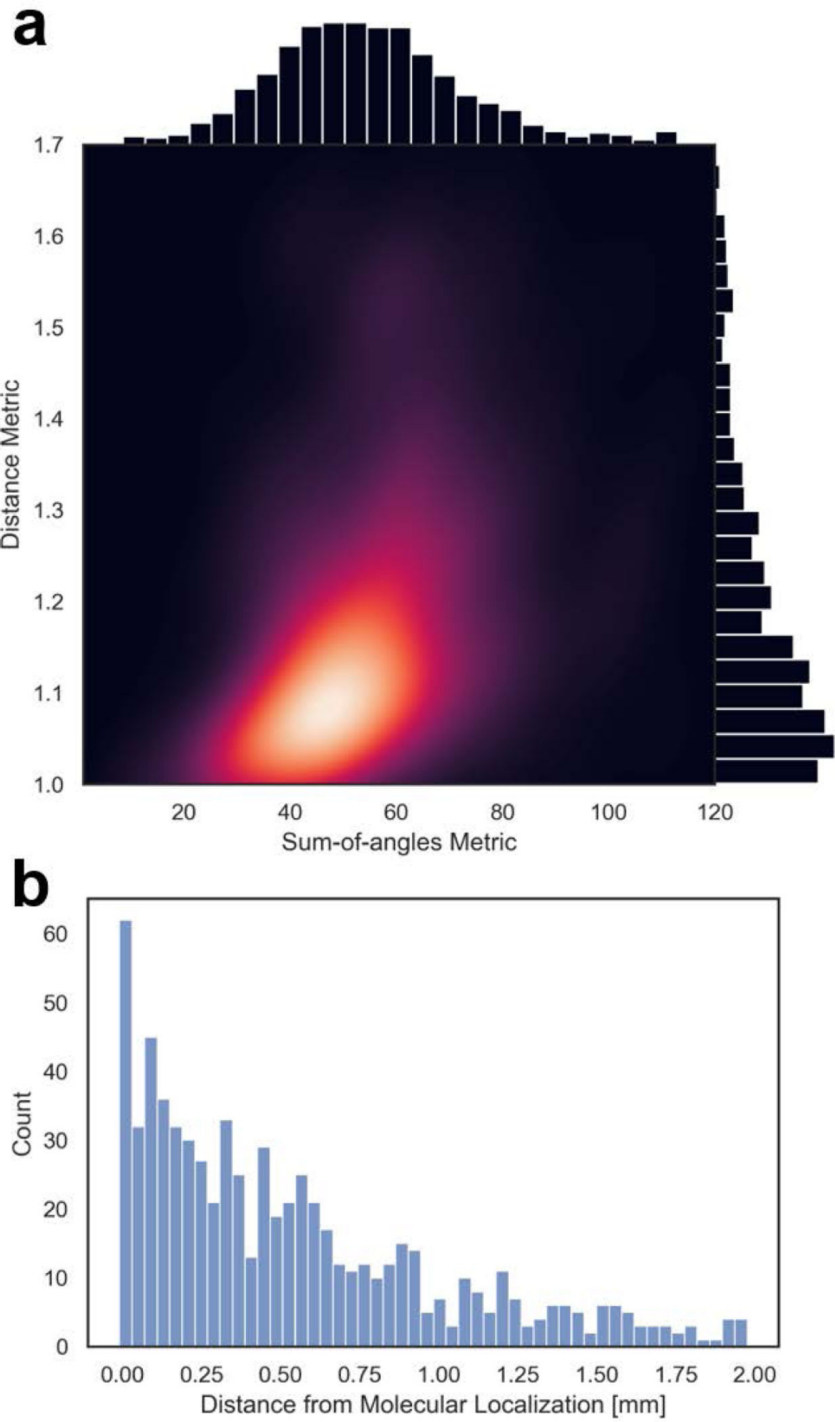
Vessel segmentation from tumor images allows for quantification of tortuosity metrics. (a) Kernel density plot of distance metric and sum-of-angles metric for all segmented vessels after removal of outliers (*n* = 698). (b) Histogram of distances between each segmented vessel and the nearest molecular localization.

**FIGURE 10.**
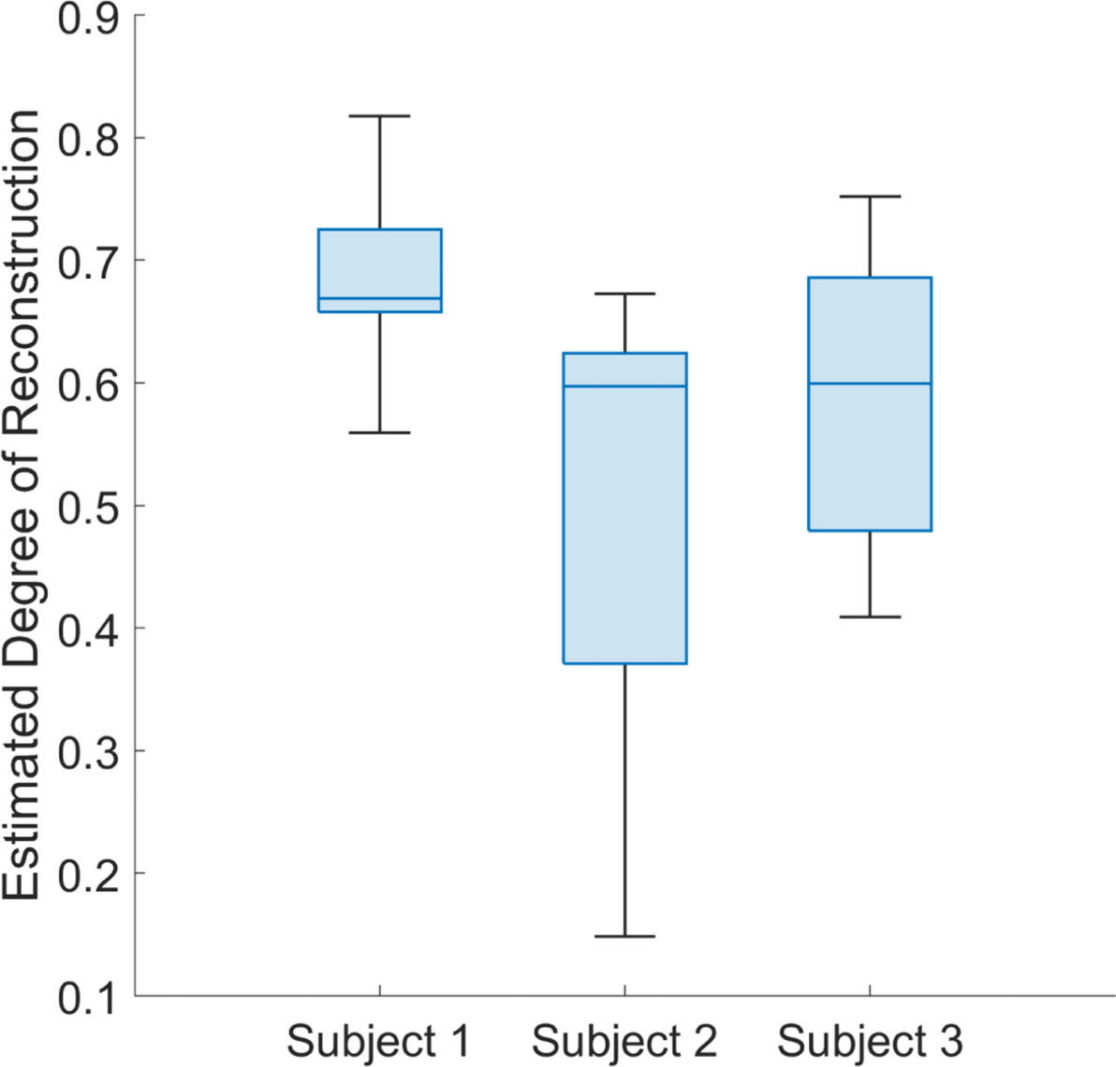
Estimated degrees of vessel reconstruction (DOR) for each tumor. Box charts show the distributions of DOR values calculated from individual slices of the tumor volumes, grouped by subject from Fig. 7. The mean DOR values in order for each tumor are 0.69, 0.51, and 0.59.

Segmentation of the vessel centerlines [38] from the ULM images allowed for quantification of tortuosity using the sumof-angles and distance metrics, both of which are elevated by malignant angiogenesis [43]. The combined distribution of DM and SOAM values for the vessels from the three tumors in Fig.7 (*n*=698 after removal of outliers) is shown in Fig.9a. From these data, we calculated $\mu_{\text{SOAM}}=54.9\pm 18.2$ and 21$\mu_{\text{DM}}=1.2\pm 0.2$. As expected, a histogram of the distances between segmented blood vessels and their nearest molecular localizations showed that the number of blood vessels decreased as the distance from molecular signaling increased. From these data, we computed $\mu_{\text{distance}}=529.7\pm 472.8\text{μ}m$ (Fig. 9b). Finally, the average estimated degrees of vessel reconstruction for each tumor from Fig. 7 in order from left to right were 0.69, 0.51, and 0.59 (Fig. 10). These values are reasonable given the duration of image acquisition and the pixel size [44].

## IV. DISCUSSION

In this study, we demonstrated a substantial advance in the resolution of USMI using high-frame-rate SpHI and MCAs targeted to angiogenic biomarkers. Concurrent optical and ultrasonic imaging in a targeted flow phantom demonstrated that this technique is sensitive to stationary bubbles and rejects flowing contrast agents. It is likely that the aggressive tracking thresholds that were used to discriminate between bound and free bubbles excluded some targeting (*e.g.*, a targeted bubble that deflates in fewer than 12 repeated pulses). However, our thresholds were tuned to minimize false positives since there are normally some residual circulating bubbles when performing ultrasound molecular imaging *in vivo*.

Furthermore, we demonstrated excellent correlation between detected ultrasound events and the ground truth microbubble density, which suggests that this method can provide superior quantification of adherent MCAs compared to standard USMI, where there are typically numerous microbubbles within a single resolution cell. As with any implementation of ultrasound localization microscopy, it is difficult to guarantee a single bubble per resolution cell at any given point in time as the bubble flow is stochastic. However, based on the linearity of the calibration results and careful control of the concentrations of the contrast media, we are confident that the majority of our localizations correspond to single bubbles.

It is important to note that the large difference in observed MCA densities between the optical and ultrasound platforms is to be expected. Because the center frequency of the receiving transducer is greater than 10× the transmit frequency, the ultrasound device is only sensitive to bubbles that produce broadband echoes. Most contrast agents will produce strong superharmonic signals when excited with sufficiently high pressures which can also result in microbubble destruction. However, at lower mechanical indices (*e.g.*, 0.24 in this study), only bubbles with resonant frequencies matching the transmit pulse will exhibit the strongly nonlinear behavior required for SpHI [27], [45], [46].Our in-house contrast agent is polydisperse, with radii ranging between approximately 0.25 and 1.5 μm. Given that the resonant frequency of a contrast agent is inversely proportional to its radius [47], only a fraction of the total distribution is matched to the transmit frequency. In the future, we may explore methods to produce more monodisperse contrast agents so that the imaging device can detect a larger percentage of the injected bubbles.

By combining molecular ULM with a motion correction scheme based on interleaved B-modes, we were able to image subcutaneous tumors in freely breathing rodents to produce co-registered maps of microvasculature and biomarker expression at a resolution beyond the diffraction limit. Previously, Shelton and colleagues demonstrated superharmonic molecular imaging *in vivo* and achieved a resolution of approximately 100 μm by transmitting and receiving at 4 and 25 MHz, respectively [42]. In the present study, a dual-frequency transducer operating at 1.7 and 18 MHz yielded a resolution of 23 μm in the same animal model used in the study by Shelton et al.

The apparent differences in perfusion between the superharmonic and ULM images in Fig. 7 can be attributed to the different methods for rendering each image (it is important to remember that they are made from the same data). The superharmonic images are maximum intensity projections, meaning that only a single contrast agent must pass through a pixel for that pixel to have a high intensity in the final rendering. It is also important to note that the PSF spans multiple pixels, so a single contrast agent will fill in a line with a thickness determined by the PSF as it flows across the imaging field. On the other hand, the ULM images are created by the superposition of individual contrast agent tracks which are typically one pixel wide. Therefore, hundreds of bubbles must be detected within a vessel for the vessel to appear with similar intensities in the final SpHI and ULM images. This is why larger vessels appear to be bright in both sets of images, whereas very small vessels containing as few as one or two contrast agents appear quite dim in comparison.

In some cases, molecular localizations appear outside of the blood vessels (Fig. 7). We believe that two factors contribute to this phenomenon, the first of which is the stochastic nature of ULM imaging. The probability that a vessel will be traversed by a contrast agent during a scan is determined by factors such as the duration of the acquisition and the concentration of the contrast agent in the bloodstream [44]. If no bubbles pass through a vessel during the scan, that vessel will not appear in the final ULM rendering. Because we are translating our linear array in the elevation dimension to interrogate the entire volume of each tumor, our total scan time is divided across the eleven scanning positions. This limits the degree of reconstruction of the vasculature, meaning that some smaller vessels do not appear in the ULM images. Using the method proposed by Dencks et al. [41], we estimated mean DOR values between 0.51 and 0.69 across the three animals (Fig. 10). The control and targeted contrast agents were administered separately, so it is likely that they sampled different subsets of the vasculature and caused some molecular localizations to appear outside of the background vessel map.

In a recent study, Hingot and colleagues empirically determined that the characteristic time of ULM reconstruction for a 2-D image of a rat brain with a continuous contrast infusion is given by *T*_*x*_ = 384*/x* seconds, where *x* is the pixel size in micrometers [44]. In the present study, *in vivo* images were reconstructed on a 10-μm grid, resulting in *T*_10_ = 38.4 seconds. The authors of [44] observed that DOR values of 0.9 were measured after acquiring data for a period of approximately 3*T*_*x*_. In our case, 3*T*_10_ = 115.2 seconds. We consider the DOR values measured from the rat tumor images to be reasonable given that we acquired data at each position for 100 seconds using a series of bolus injections rather than a continuous infusion.

Two ways to improve the degree of reconstruction without compromising the resolution are (1) increasing the contrast agent dose and (2) increasing the imaging duration at each location. With the current imaging system, the contrast agent dose is optimized. That is, it cannot increase without image degradation due to overlapping bubbles during the localization process. We also cannot increase scanning time because of limitations imposed by our animal protocol related to anesthesia. Therefore, we believe that the best way to improve DOR in future studies (and hence overlap between the molecular localizations and the background vessels) is to replace the linear array with a volumetric imaging system so that the entire tumor can be interrogated simultaneously. In our case, this would increase the scan time at each voxel by a factor of 11, since we have 11 different imaging positions.

The second factor contributing to seemingly erroneous molecular localizations is motion correction. The speckle pattern of the high-frequency linear array decorrelates quickly with elevational displacements (*e.g.*, from respiratory motion) which reduces the accuracy of in-plane motion tracking and the final image registration. A matrix array designed for SpHI would allow us to beamform isotropic voxels for robust elevational tracking and simplified image registration. Additionally, correcting the disparity between the spatial sampling frequencies of the different dimensions would lead to an improved representation of vessel positions and statistics. We believe that the molecular localizations that are near, but not within, a vessel would be matched with a vessel if it were possible to eliminate the error introduced by physiological motion over the course of the long acquisition time.

Motion might also increase the number of false negatives in the molecular images. Consider the case where the molecular scan for a particular position begins as the animal is taking a breath; targeted bubbles in the field of view will likely be destroyed by the repeated transmissions by the time the breath has concluded. Any frames containing the bound bubbles will be discarded because of the large tissue displacements, and so these contrast agents will not appear in the ULM images. This is one possible reason that Subject 1 appears to have fewer binding events than the other two animals (Fig. 7j), though this could also be the result of inter-animal variability. In future work, we plan to implement a motion gating system to ensure that our acquisitions are not adversely affected by breathing and other sources of tissue motion.

As mentioned previously, we were unable to increase the contrast agent dose without experiencing ULM image degradation from overlapped microbubble signals in the beamformed images. Recent studies have introduced a variety of methods for improving localization accuracy for very high concentrations of microbubbles. In [48], the authors used a deep convolutional neural network (CNN) to map low-resolution CEUS images to sparse, super-resolved images of microbubble positions. They observed that the CNN outperformed a standard localization procedure for higher microbubble concentrations. Milecki and colleagues extended this approach by training an encoder-decoder network to map spatiotemporal data (*i.e.*, sets of subsequent CEUS images) to single images of super-resolution microbubble paths [49]. Most recently, Blanken et al. demonstrated super-resolution imaging of high-density microbubble clouds by performing deconvolution in the channel data space with a 1-D CNN [50]. In [51], the authors applied overlapping bandpass filters to sets of CEUS images in the 3-D Fourier space to retrieve sparse subsets of the total microbubble population which could be localized with traditional means. In future work, we will explore various techniques such as these to increase the microbubble dose and degree of vessel reconstruction.

Despite the aforementioned limitations, the results presented herein constitute a compelling argument for the feasibility of super-resolution USMI. The aim of the present study is not to draw any biological conclusions based on the results, but rather to demonstrate the capabilities of superharmonic imaging for imaging stationary contrast agents *in vivo* so that they may be localized and processed. We believe that this new imaging method has the potential to significantly augment the capabilities of ULM and molecular imaging for quantifying features of the tumor microenvironment in preclinical and clinical studies alike.

For example, we demonstrated that we can perform image analysis with a greater degree of granularity and describe the relationships between individual capillaries and points of molecular signaling. With these data, it is now possible to study the effects of molecular signaling on the local architecture of small blood vessels by investigating how measures of vessel density, radius, and tortuosity are affected by proximity to points of molecular targeting. While the scope of the present study is limited to the description and validation of the new imaging method, in future work we plan to compare the proposed technique with traditional USMI for applications such as monitoring response to tumor treatment.

## V. CONCLUSION

We present a proof-of-concept study for super-resolution ultrasound molecular imaging with superharmonic imaging. *In vitro*, we demonstrate that it is possible to detect and localize stationaryultrasound contrast agents using superharmonic imaging, and we validate the method with high frame-rate optical microscopy. In a rat fibrosarcoma model, we measure a resolution *in vivo* of 23 μm, a significant improvement compared to previous work. We also propose a variety of approaches to quantification which are currently not possible with diffraction-limited imaging techniques. In future studies, we plan to investigate how vessel statistics change in response to proximity to molecular signaling. Furthermore, we also hope to study how molecular signaling and vessel structure respond to interventions such as radiotherapy or anti-angiogenic treatments.

## Figures and Tables

**TABLE 1.** Summary of the ultrasound contrast agents used in the present study.

Contrast agent	Binding target	Mean diameter	Standard deviation	Stock concentration

Control	N/A	1.67 μm	1.10 μm	1.0e10 mL^−1^
cRGD	$\alpha_{v}\beta_{3}$ integrin	1.51 μm	0.92 jam	5.6e9 mL^−1^
Biotin	Avidin	1.21 μm	0.76 μm	3.3e10 mL^−1^
